# The role of cilia during organogenesis in zebrafish

**DOI:** 10.1098/rsob.230228

**Published:** 2023-12-13

**Authors:** Junjun Liu, Haibo Xie, Mengfan Wu, Yidan Hu, Yunsi Kang

**Affiliations:** ^1^ College of Marine Life Sciences, Ocean University of China, Qingdao 266003, People's Republic of China; ^2^ Institute of Evolution & Marine Biodiversity, Ocean University of China, Qingdao 266003, People's Republic of China

**Keywords:** cilia, organogenesis, zebrafish, ciliopathies

## Abstract

Cilia are hair-like organelles that protrude from the surface of eukaryotic cells and are present on the surface of nearly all human cells. Cilia play a crucial role in signal transduction, organ development and tissue homeostasis. Abnormalities in the structure and function of cilia can lead to a group of human diseases known as ciliopathies. Currently, zebrafish serves as an ideal model for studying ciliary function and ciliopathies due to its relatively conserved structure and function of cilia compared to humans. In this review, we will summarize the different types of cilia that present in embryonic and adult zebrafish, and provide an overview of the advantages of using zebrafish as a vertebrate model for cilia research. We will specifically focus on the roles of cilia during zebrafish organogenesis based on recent studies. Additionally, we will highlight future prospects for ciliary research in zebrafish.

## Graphical abstract

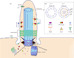

## 1. Introduction

Cilia are evolutionarily conserved organelles that are widely present in most types of human cells [1]. Cilia play a crucial role in the normal development of human embryos, including organ morphogenesis and the establishment of left–right asymmetry [2]. In adults, cilia are also involved in various biological processes, such as maintaining the morphology and function of photoreceptors and contributing to kidney homeostasis. Abnormal ciliary function in humans is associated with a range of diseases, including situs inversus, polycystic kidney disease, polydactyly, blindness, mental retardation and obesity. These conditions are collectively known as ciliopathies [3]. Ciliopathies are genetic disorders in humans caused by defects in cilia formation or dysfunction, typically inherited as recessive traits [4]. Ciliopathies are characterized by genetic heterogeneity and phenotypic variability. For instance, mutations in certain ciliary genes can cause multiple types of ciliopathies, and different gene mutations can lead to the same ciliopathy [3,5]. Consequently, predicting the clinical phenotype resulting from specific gene defects remains a significant challenge. Currently, only a small number of genotypic and phenotypic associations have been clinically elucidated in patients. Therefore, studying the structure and function of cilia can provide a theoretical basis for understanding the pathogenic mechanisms underlying ciliopathies.

Over the past few decades, studies from *Chlamydomonas* and *Caenorhabditis elegans* have provided significant insights for our understanding of the mechanisms of ciliogenesis [6,7]. Noticeably, these invertebrate models are limited by their underdeveloped organs, such as eyes and kidneys, which hinder their ability to accurately replicate organ abnormalities observed in human ciliopathies. In recent years, vertebrate models including mice, zebrafish and *Xenopus* have demonstrated significant advantages in investigating the physiological functions of cilia and ciliopathies [8–10]. Among these vertebrate models, zebrafish uniquely possess several key advantages. These include higher spawning capacity and a consistently stable spawning period, *in vitro* fertilization and development, embryonic transparency, mature and diverse genetic manipulation techniques, and high-resolution live imaging. Moreover, zebrafish organs display anatomical similarities to those of humans, such as the eyes, kidneys and spine. These distinctive characteristics of zebrafish offer new perspective into understanding the physiological roles of cilia and the pathogenesis of ciliopathies.

In this review, we will first outline the structure and function of cilia. Secondly, we will introduce the different types of cilia present in zebrafish and summarize the advantages of studying cilia using this model. Next, we will focus on recent advances in understanding the physiological functions of cilia during zebrafish organogenesis. This includes exploring the roles of cilia in left–right asymmetry determination, photoreceptor maintenance, kidney development, the homeostasis of the body axis and spine development, as well as gametogenesis and fertilization. Finally, we will highlight current opportunities for cilia research using the zebrafish model, providing a reference for future investigations.

## 2. Structure of cilia

**Figure 1.**
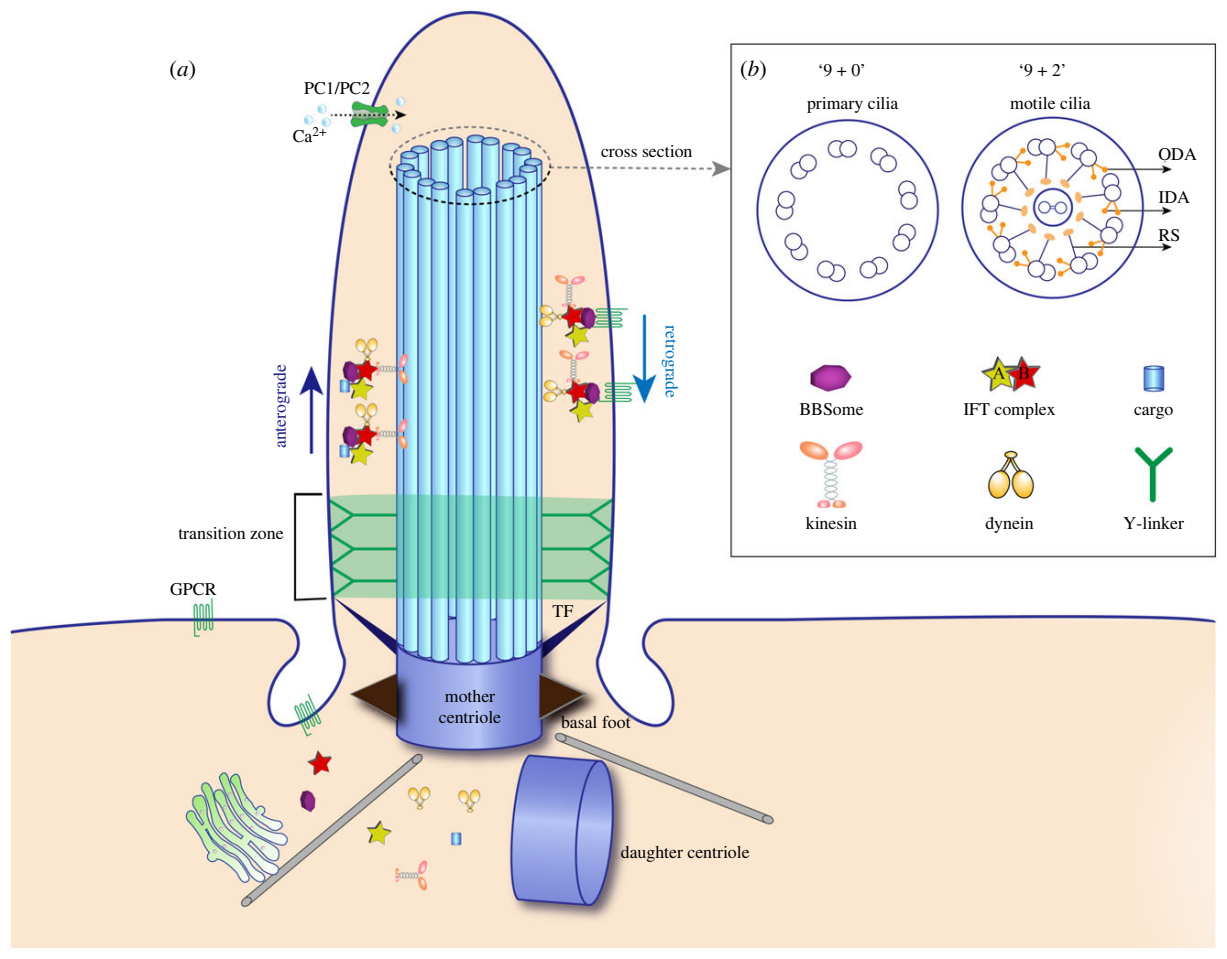
Schematic structure of cilia. (*a*) The basic structure of cilia. Cilia extend from the basal body, which is composed of a mother centriole and distal appendages including transition fibres that connect the cilia with cell membrane. The transition zone is a structural intermediate between the basal body and axoneme that plays an essential role in controlling the entry/exit of ciliary proteins. The transition zone is enriched in Y-linkers that connect the axoneme with ciliary membrane. The ciliary membrane is enriched in signal receptors and ion channel proteins. The core structure of cilia is composed of nine doublet microtubules. Anterograde kinesin-2 and retrograde dynein motors drive the bidirectional transport of IFT trains. (*b*) Diagram of the cross section of cilia. Primary cilia are characterized by a typical ‘9 + 0’ architecture. Motile cilia are characterized by a canonical ‘9 + 2'architecture, which consist of nine doublet microtubules surround a central pair of singlet microtubules. The motility of cilia relies on inner/ outer dynein arms and radial spokes. Abbreviations: TF, transition fibre; ODA, outer dynein arms; IDA, inner dynein arms; RS, radial spoke; GPCR, G-protein coupled receptor; PC1/2, polycystin-1/2.

Cilia are hair-like protrusions that originate from the cell surface, covered by a double-layered lipid membrane and derived from the mother centriole of the basal body [11]. Cilia are highly conserved organelles and are widely present from unicellular organisms to humans. The structure of cilia consists of the basal body, transition zone and axoneme, covered by the ciliary membrane, which contains signalling receptors and ion channel proteins [5,12,13] (figure 1). The basal body, located at the base of the cilium, is composed of nine triplet microtubules. These triplet microtubules extend outward to form doublet microtubules, which constitute the axoneme [14–16]. The transition zone, situated between the basal body and axoneme, acts as a barrier that controls the entry and exit of ciliary proteins, contributing to the maintenance of the internal environment within cilia. Transition zone is characterized by numerous transition fibres and Y-linkers. Transition fibres connect the basal body to the base of the ciliary membrane, while Y-linkers connect the doublet microtubules to the ciliary membrane [17–19]. The axoneme, protruding from the cell surface, is the main part of the cilium. It generally consists of nine doublet microtubules derived from the basal body, with each doublet microtubule composed of a complete microtubule (A-tubule) and an incomplete microtubule (B-tubule) [20].

Protein synthesis does not occur within the cilium; instead, the assembly, maintenance and signal transduction of the cilium are dependent on intraflagellar transport (IFT). IFT is a protein complex that transports cargo bi-directionally within the microtubule axoneme, forming IFT trains [21,22]. IFT particles are composed of IFT-A and IFT-B complexes. The IFT-A complex consists of six proteins, while the IFT-B complex consists of 16 proteins and can be further divided into IFT-B1 (10 subunits) and IFT-B2 (6 subunits) [21,23,24].

Anterograde transport of IFT, driven by kinesin-2, carries proteins along the B-tubules from the base to the tip of the cilium, and cargo is released from IFT at the ciliary tip [25,26]. Subsequently, retrograde transport of IFT, driven by dynein-2 along the A-tubules, returns to the base of the cilium [27,28]. Two types of kinesin-2 motors are involved in the anterograde transport of IFT: a heterotrimer composed of KIF3A, KIF3B/KIF3C and KAP, and a homodimer composed of KIF17. Additionally, another important protein complex called BBSome proteins plays a crucial role in regulating the entry and exit of membrane proteins [29–31].

## 3. Classification and function of cilia

Cilia can be classified into two types based on their motility: motile cilia and primary cilia. Motile cilia have a ‘9 + 2’ structure, consisting of nine doublet microtubules around a central pair of single microtubules. By contrast, primary cilia (also known as non-motile cilia) have a ‘9 + 0’ structure, composed of nine doublet microtubules without the central pair [11]. Motile cilia possess radial spokes, inner and outer dynein arms, which are absent in primary cilia. Radial spokes connect the doublet microtubules to the central microtubules, while the inner and outer dynein arms extend from the A-tubule to the B-tubule. The inner and outer dynein arms provide the power for ciliary movement, with the outer dynein arm driving ciliary beating and the inner dynein arm regulating the pattern of beating. The cross-sectional view of the axoneme exhibits a wheel-like structure that supports both motility and structural integrity [32–34]. However, it is important to note that the type of ciliary structure does not always strictly correspond to its motility. For instance, the cristae in the otic vesicle of zebrafish are immotile cilia with a ‘9 + 2’ structure, while the cilia in the node of mouse embryos and Kupffer's vesicle (KV) of zebrafish are motile cilia with a ‘9 + 0’ structure [35–37].

Motile cilia drive the movement of extracellular fluids and serve various functions, including cellular movement, signal transduction and waste clearance. In the reproductive system, ciliary motility is crucial for the movement of sperm and fertilized eggs. Mature sperm swim using their flagella to facilitate the process of fertilization, while the migration of fertilized eggs to the uterus relies on cilia in the fallopian tubes. Ciliary defects in the movement of sperm or fallopian tube can lead to infertility or sterility [38,39]. Motile cilia in the ventricles and central canal promote the flow of cerebrospinal fluid, which contains signalling molecules necessary for organism growth and development. Therefore, defects in ciliary beating in these areas can cause abnormal cerebrospinal fluid circulation, resulting in hydrocephalus [40–42]. The rhythmic beating of cilia in the node of the mouse and KV of zebrafish creates a polarized distribution of signalling molecules and ensures the proper distribution of organs [35,43]. Coordinated ciliary beating in the respiratory tract helps clear mucus that has trapped dust particles and pathogens, serving as the first line of defense against pathogens [44,45].

Primary cilia, on the other hand, function as sensors that accept, process and transmit signals related to development, physiology and the environment. Primary cilia are involved in the transmission and formation of signals in human visual, auditory and olfactory senses. Primary cilia membranes contain receptors and ion channel proteins, and the transmission of multiple signalling pathways depends on primary cilia [5]. In the visual system, the outer segment of photoreceptors (cone and rod cells) is a specialized cilium that contains opsin, a protein that detects light signals. Photoreceptors process light signals and transmit them to the brain, resulting in vision [46]. Olfactory receptors are distributed on the cilia of olfactory neurons. When odour molecules bind to these receptors, the olfactory signal cascade is activated, and the signal is transmitted to the brain, producing the sense of smell [47,48]. The kinocilium in the ear can sense the vibrations of sound waves and transmit these signals to the central nervous system, contributing to hearing [49]. Furthermore, primary cilia are involved in multiple signalling pathways, including Wnt, Hedgehog, Notch, planar cell polarity (PCP) and TGF-β [50]. Among these, the Hedgehog signalling pathway is the most well known and plays a crucial role in embryonic development and tissue homeostasis. In the absence of Sonic Hedgehog (SHH), Patched (PTCH) accumulates on the ciliary membrane, inhibiting the aggregation of Smoothened (SMO) in the cilium. When SHH binds to PTCH, the inhibition of SMO is released, allowing SMO to accumulate on the cilium and promote the translocation of GLI proteins into the nucleus to regulate the expression of Hedgehog signalling-related target genes [51–53].

## 4. Cilia in zebrafish

Zebrafish have been used as an animal model for genetic research since the early 1980s [54]. Zebrafish play a significant role in the study of early embryonic development and human diseases [9]. The zebrafish genome is approximately 1.4 Gb in size, distributed across 25 chromosomes and encodes around 26 000 proteins. It exhibits an 87% similarity with the human genome, with more than 82% of human pathogenic genes having homologues in zebrafish [55]. Zebrafish is a major model for live imaging in vertebrates, owing to its *in vitro* development and transparent embryos [9,56–58]. In terms of genetic manipulation, forward genetics techniques are commonly employed in zebrafish to screen mutants through radiation, chemical reagents and DNA insertion, enabling genetic research in embryonic development and organ formation [59]. Currently, gene editing methods such as ZFNs, TALENs and CRISPR/Cas9 have become the main tools for gene function research in zebrafish [60–66]. Morpholino-mediated gene knockdown is also extensively used in zebrafish research. Additionally, the Tol2 transposon system has been employed for the construction of transgenic zebrafish [67,68]. The GAL4/UAS and Cre/LoxP systems are used for temporal and spatial control of gene expression and conditional gene knockout by coupling with regulatory elements [69–72].

**Figure 2.**
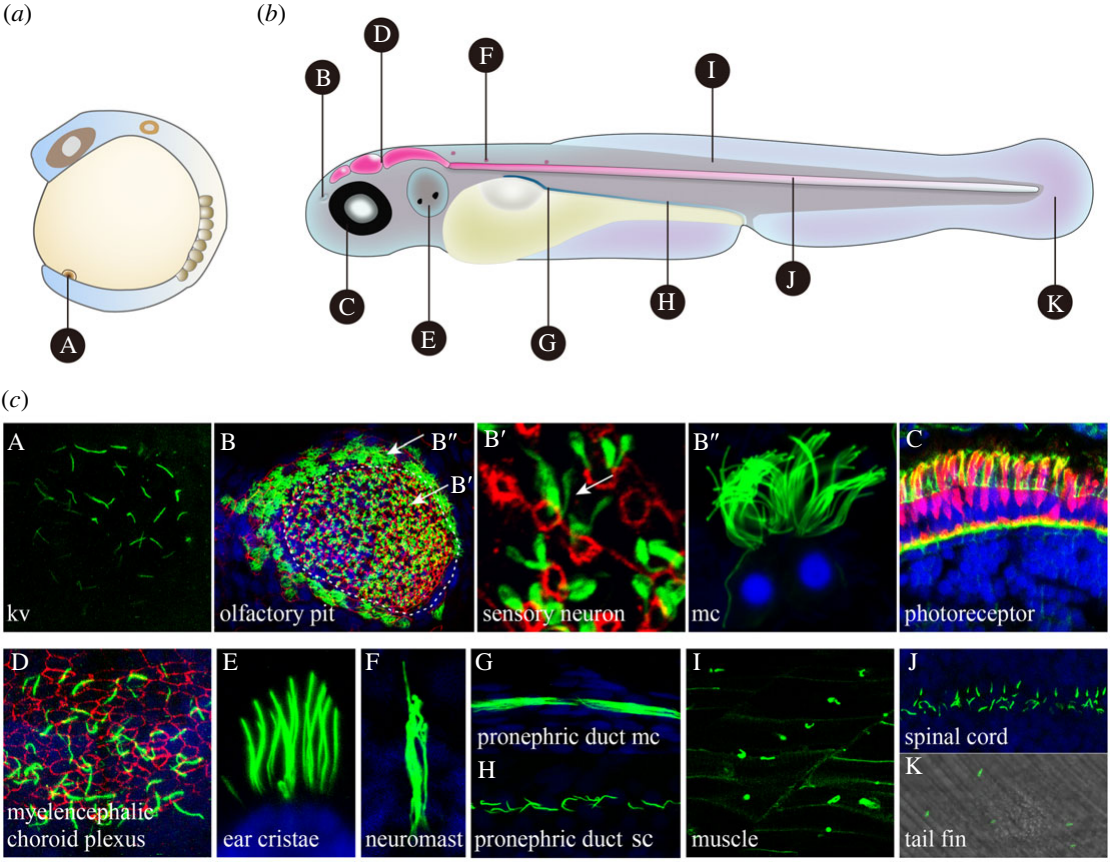
Cilia in zebrafish larvae. (*a*,*b*) Diagrams indicate the position of panels A–K where the corresponding cilia were obtained. (*c*) Panel A shows the cilia in the KV; panels B–B″ indicates the cilia in the olfactory pit at 5 dpf, B′ and B″ refer to the cilia indicated by the arrows in (*b*). Tight junction was stained with Claudin-5 antibody in red. Confocal images of transverse section in C show the photoreceptor cells of zebrafish at 5 dpf. Actin filaments were stained with phalloidin in green, images showing the cell body of double cone photoreceptors visualized by ZPR-1 antibody in red. Panel D shows the cilia in myelencephalic choroid plexus. Tight junction was stained with Claudin-5 antibody in red. Confocal images showing the cilia in the ear cristae (E), neuromast (F), the anterior part of pronephric duct enriched with multicilia (G), the posterior part of pronephric duct enriched with single cilia (H), and spinal cord (J) at 5 dpf. Panels I and K show the images of *Tg(bactin2:: Arl13b-GFP)* transgenic embryos, revealing cilia in muscle cells (I), and cilia in skin cells of the tail fin (K). Cilia in A were labelled with anti-acetylated tubulin antibody. Cilia in B–B″, D–H and J were labelled with anti-mono-glycylated tubulin antibody. In all panels, blue represents the nuclei, labelled with DAPI. Abbreviations: mc, multicilia; sc, single cilia.

**Figure 3.**
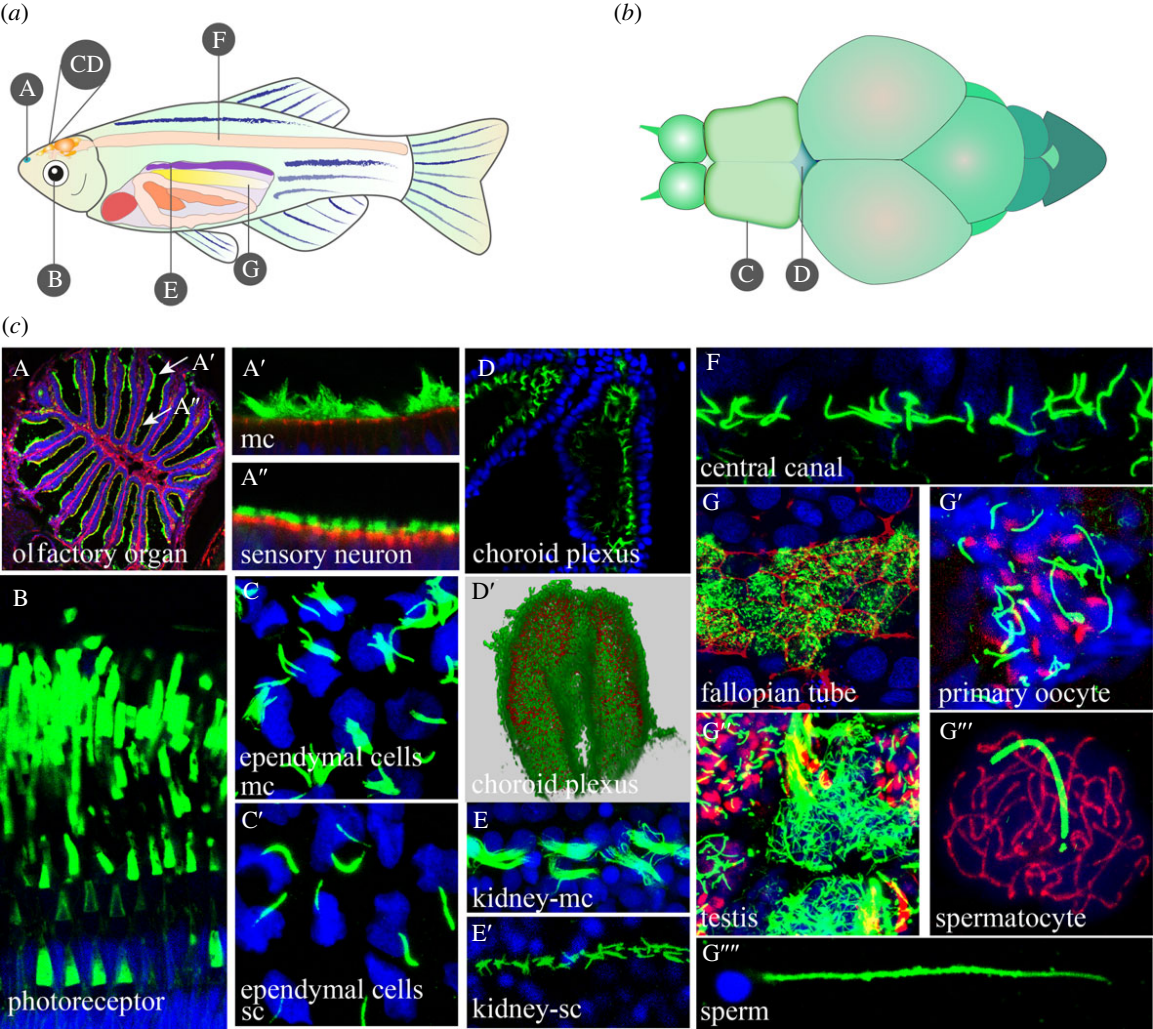
Cilia in adult zebrafish. (*a*) Diagram indicating the position in panels A–G where the corresponding cilia were obtained. (*b*) Diagram showing the position in adult brain tissue where the cilia were obtained. (*c*) Confocal images in panels A–A″ show cilia in the olfactory organ. A′ indicates the multicilia in the periphery epithelial cells. A″ indicates the cilia in the olfactory sensory neuron in the centre region. Confocal images of transverse section in B show the photoreceptor cells of zebrafish. The outer segment of photoreceptor was stained with WGA in green. In C–C′, confocal images showing multicilia (C) and single cilia (C′) of ependymal cells. In D-D′, images showing multicilia of diencephalic choroid plexus (dChp). In D, a dorsal view of diencephalic choroid plexus; In D′, the diagram of three-dimensional reconstruction of the diencephalic choroid plexus. Cilia were visualized with anti-gly-cylated tubulin in red, nuclei were counterstained with DAPI in green. In E,F, confocal images show the multicilia (E) and single cilia (E′) in the kidney, and cilia in the central canal (F). In G–G*′′′′*, confocal images show the cilia in the reproductive system as indicated in fallopian tube (G), primary oocyte at one month old (G′), the testis (G″), the spermatocyte (G′′′) and the sperm (G′′′′). The red shows the F-actin stained by phalloidin (G), the double-strand breaks stained by γ-H2A.x (G′–G*″*) and the chromosome stained by SYCP3 antibody (G′′′). Cilia in A–A″, G-G′′′′′ were labelled with anti-acetylated tubulin antibody. Cilia in C–C′, D–F were labelled with anti-mono-glycylated tubulin antibody. In all panels, blue represents the nuclei, labelled with DAPI. Abbreviations: mc, multicilia; sc, single cilia.

Zebrafish offer several advantages for ciliary research in vertebrates. Firstly, like humans, cilia are present in almost all cells of zebrafish (figures 2 and 3). Secondly, the types and structures of cilia in zebrafish are diverse as well. Multicilia are mainly found in the olfactory organ, pronephric/kidney duct, ependymal cells and fallopian tubes, while motile cilia are primarily distributed in the early otic vesicle, KV, brain ventricles, central canal, pronephric/kidney duct, fallopian tubes and periphery of olfactory organs. In addition to these types of cilia, there are immotile multi-cilia in olfactory neurons as well as single immotile cilia in KV and other tissues (figures 2 and 3) [37,73–76]. Most motile cilia in zebrafish exhibit an ultrastructure of ‘9 + 2’, but there are ‘9 + 2’ or ‘9 + 0’ motile cilia in the neural tube and KV [37,76]. Most primary cilia exhibit an ultrastructure of ‘9 + 0’, while the kinocilia in the otic vesicle and cilia in the olfactory neuron are ‘9 + 2’ immotile cilia [36,77–79]. Moreover, the pattern of ciliary beating in zebrafish is also diverse. Motile cilia in the KV and early otic placode exhibit a circular beating pattern [80]. In the pronephric/kidney duct, motile cilia show a rhythmic sinusoidal wave pattern, while in the central canal and ventricles, the beating pattern is back and forth [42,73].

Furthermore, the anatomical similarities between zebrafish and humans in terms of tissues and organs make it a reliable model for simulating the physiological functions of cilia in humans [9,81]. Current research demonstrates that cilia in the KV (resembling the nodal cilia in mammalian embryos) contribute to the formation of left–right asymmetry in internal organs. Cilia in the otic placode play a role in sensing sound waves, while cilia in the ventricle and central canal facilitate the flow of cerebrospinal fluid, crucial for maintaining the development of the body axis. Cilia in the pronephric duct aid in urine excretion, and the lateral cilia can perceive various water flow factors. Additionally, cilia can act as cellular antennae for sensing environment signals in photoreceptors and other neurons [74,75]. Interestingly, recent studies by our group and Mytlis *et al*. have discovered that cilia are also present in primary spermatocytes and oocytes, where cilia may participate in the regulation of meiosis [82,83].

## 5. Cilia and organogenesis in zebrafish

### 5.1. Recent advances of cilia research based on zebrafish

**Figure 4.**
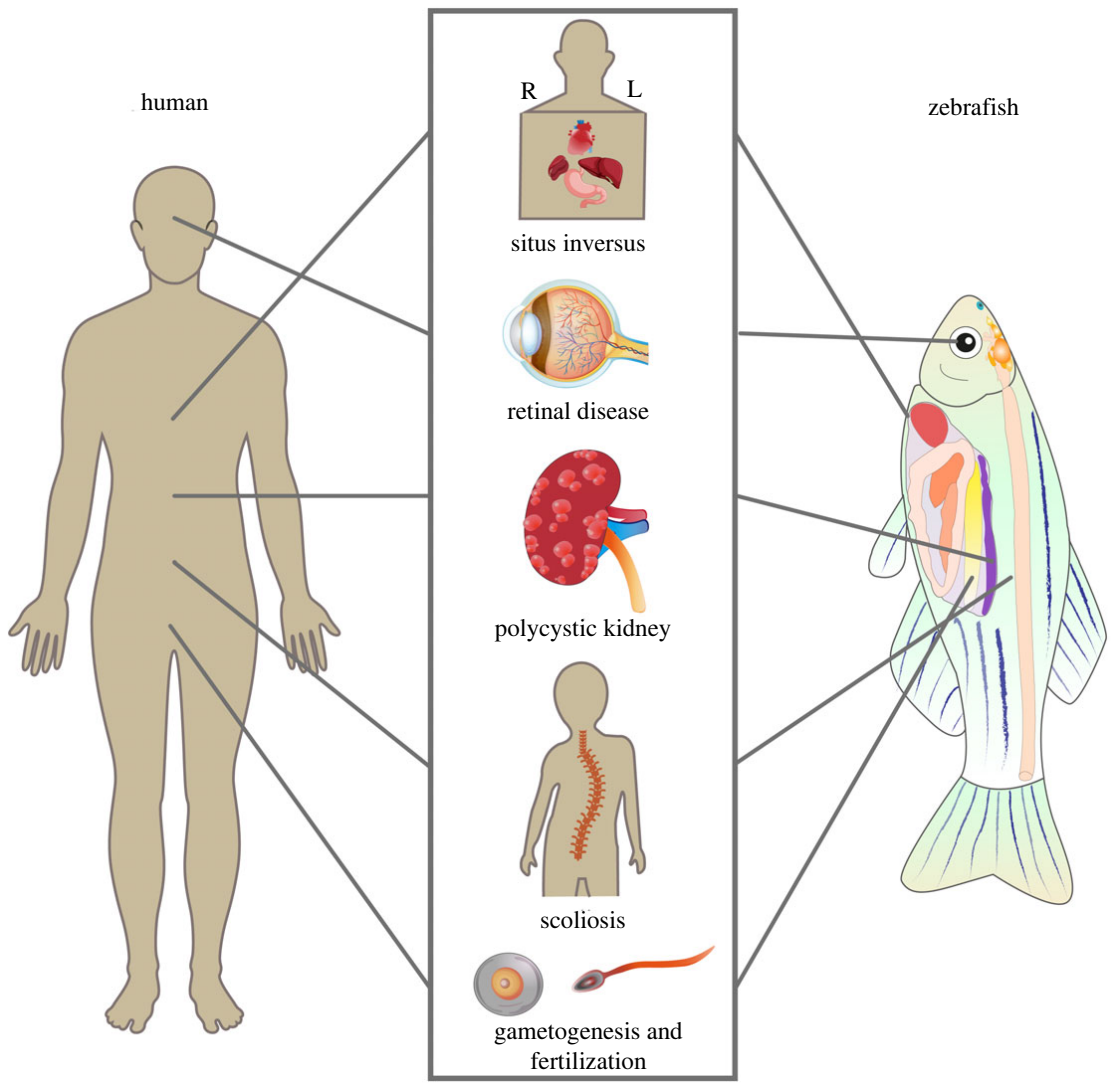
Recent progress on physiological function of cilia based on zebrafish. Zebrafish is an ideal model to study human disease caused by ciliary dysfunction, including abnormal asymmetry pattern of internal organs, retinal diseases, polycystic kidney, scoliosis, gametogenesis and fertilization.

Recently, significant progress has been made in various areas of cilia research using zebrafish as a model organism. These areas include understanding the structure of cilia, ciliogenesis, the physiological functions of cilia, signalling transduction within cilia and human diseases caused by ciliary defects. Here, we will summarize recent advance on the roles of cilia during zebrafish organogenesis by focusing on the left–right asymmetry determination, photoreceptor homeostasis, kidney function, body axis development and gametogenesis (figure 4).

### 5.2. Cilia and left–right asymmetry

During the early development of vertebrate embryos, the correct establishment of asymmetrical arrangement of internal organs is crucial. In humans, an incorrect left–right asymmetry of internal organ development can lead to a series of birth defects, including congenital heart defects, bilateral or trilobar lung malformations, defects in visceral connections and visceral ectopia. Visceral ectopia can also cause heterotaxy syndrome, a genetic disorder that affects approximately 1 in 10 000 people at birth and is primarily characterized by the randomization of internal organ positions. The primary health issue for heterotaxy patients is congenital heart disease [84]. Patients with primary ciliary dyskinesia (PCD) often exhibit left–right asymmetrical defects, such as visceral ectopia or complete ectopia, which are primarily caused by ciliary motility defects [85,86].

The determination of left–right asymmetry is governed by a transient organ known as the left–right organizer (LRO). In mice, it is referred to as the ventral node, Hensen's node in chickens, KV in zebrafish and the gastrocoel roof plate in *Xenopus* [37,87–89]. Primary cilia and motile cilia in the LRO play a crucial role in generating and transducing the directional flow of extracellular signals into asymmetric nodal signalling, which influences organ laterality [90]. Motile cilia in the LRO generate leftward fluid flow, while primary cilia detect this flow. As a result, calcium ions in the left cytoplasmic mesoderm are rapidly transferred to the surrounding tissues, leading to the downregulation of the nodal repressor *DAND5* on the left side of the LRO and increased expression of *DAND5* on the right side [88,91]. This asymmetrical expression of nodal signals activates the nodal negative feedback inhibitor Lefty [88,92,93]. The asymmetric nodal signal also triggers the expression of other genes, such as *PITX2*, on the left side, which contributes to asymmetric morphogenesis of internal organs, including the heart, lungs and kidneys [88,94,95]. Recently, a publication in evolutionary developmental biology identified a metalloprotease known as ‘ciliated left right organizer metallopeptidase’ (CIROP) and demonstrated its specific expression in LRO cells. This study found that CIROP serves as an upstream factor essential for DAND5 asymmetry in fish, frog and mouse embryos. Moreover, CIROP mutations were discovered in humans with autosomal recessive heterotaxy. The effects of fluid dynamics within the LRO on CIROP and the specific mechanism by which CIROP influences DAND5 remain unclear [96]. However, several questions remain regarding the establishment of left–right asymmetry in vertebrates, particularly regarding how cilia sense fluid flow in the LRO. Two hypotheses have emerged: (1) cilia sense the morphogens carried by the fluid flow [97]; and (2) cilia detect the mechanical force exerted by the fluid flow [90].

Numerous studies have demonstrated that the mechanism of asymmetric establishment mediated by KV in zebrafish is similar to that in humans. In zebrafish, mutations in genes associated with motile cilia often result in left–right asymmetry defects, and the ability to culture zebrafish embryos *in vitro* makes the study of the LRO more convenient [9,98]. Recent studies in zebrafish have provided valuable insights into the role of cilia in sensing fluid flow in the LRO. These studies support the hypothesis that cilia function as calcium-mediated mechanical sensors, regulating left–right asymmetry. In 2015, Yuan *et al*. [99] developed transgenic zebrafish that specifically indicated intraciliary calcium signals. They discovered that intraciliary calcium oscillations at the LRO, coinciding with the onset of LRO ciliary motility, occurred earlier than the known left–right asymmetry signalling molecules [99]. The intraciliary calcium oscillations at the LRO significantly decreased in *pkd2* morphants or *pkd2* mutants, which also displayed random distribution of hearts. This finding suggests that the ciliary calcium oscillations depend on Pkd2 and are necessary for establishing left–right asymmetry [99]. However, due to technical limitations, it has been challenging to determine whether cilia in the LRO act as mechanosensors or chemosensors that mediate these asymmetric calcium ion signals *in vivo*.

More recently, Yuan *et al*. developed a research platform called CiliaSPOT, which combines light sheet microscopy and optical tweezers. This platform enables the manipulation of cilium bending to simulate fluid flow. The dynamic changes of calcium ions captured by the light sheet microscope were analysed using CiliaNet, a deep-learning working platform. By using CiliaSPOT to trap and bend cilia in the LRO of *c21orf59* knockdown embryos, intraciliary calcium oscillations were observed. By contrast, intraciliary calcium oscillations were not observed in *pkd2* morphants or *pkd2* mutants when cilia were mechanically bent using CiliaSPOT. These findings suggest that LRO cilia are mechanosensitive and convert mechanical forces into calcium signals through the Pkd2 cation channel to regulate left–right asymmetry [58]. Independently, working in mouse, Katoh *et al*. found that immotile cilia at the nodal sense the mechanical force generated by the flow [100].

### 5.3. Cilia and photoreceptor

Photoreceptors are specialized sensory neurons responsible for transforming light stimuli into electrical signals, facilitating the creation of visual perceptions. Photoreceptors consist of outer segment (rich in rhodopsin), inner segment, cell body and synapses [101]. Photoreceptors can be categorized into two types: rods and cones, named for their distinctive morphology. Rods have cylindrical-shaped outer segments, while cones have conical-shaped outer segments. The outer segment is a highly specialized primary cilium. The outer segment and inner segment are connected by a connecting cilium (CC), which consists of nine double microtubules and is analogous to the transition zone of the primary cilium [101,102]. Abnormal development, dysfunction or degeneration of photoreceptors can result in a range of hereditary retinal diseases. There are many human diseases caused by photoreceptor dysfunction, including retinitis pigmentosa, Leber congenital amaurosis (LCA), and rod and cone dystrophy [103,104]. More than 200 genes have been predicted to be involved in photoreceptor degeneration, and approximately one-third of these genes have been reported to be associated with defects in IFT.

Unlike rod-dominant retinas in amphibians or rodents, the types and proportions of photoreceptors in zebrafish are highly similar to those in humans. Approximately 10% of the outer segment is renewed every day [105,106]. Since the outer segment lacks protein synthesis machinery, all proteins of the outer segment must be synthesized in the inner segment and transported to the outer segment through IFT. Therefore, protein trafficking in the CC is essential for the development and maintenance of photoreceptors [107,108]. Studies in zebrafish have shown that dysfunction of IFT transport affects the genesis or maintenance of photoreceptor outer segments. Mutations in *ift52*, *ift57* and *ift88* (components of the IFT-B complex) result in the absence of outer segments, followed by the degeneration of photoreceptors [109,110]. Further studies have revealed that *ift57* mutants form short outer segments but fail to maintain them, leading to rapid photoreceptor degeneration [110,111]. Both *ift74* morphants and *ift74* mutants exhibit outer segment degeneration [112,113]. *Ift80* morphants fail to form outer segments, ultimately resulting in the apoptosis of photoreceptors [114]. *Ift122* mutants display a slow onset of progressive photoreceptor degeneration after 7 days post-fertilization (dpf) [115]. *Ift88* and *ift172* mutants are unable to form the outer segment of photoreceptors and exhibit rapid photoreceptor degeneration [111].

The BBSome complex plays a crucial role in regulating IFT, and mutations in BBSome genes are often associated with retinitis pigmentosa in patients [104,116,117]. In zebrafish, mutation of BBSome genes leads to photoreceptor degeneration [112,113,118,119]. Specifically, knocking down the vision-specific transcript *bbs3l* in zebrafish contributes to impaired visual function and mislocalization of green cone opsin [118]. BBS2 is a protein associated with the basal body that is essential for ciliogenesis. Mutations in BBS2 result in retinal degeneration [120,121]. Zebrafish *bbs2* mutant larvae exhibit impaired visual function in optokinetic response (OKR) experiments, and adult *bbs2* mutants undergo progressive degeneration of cone photoreceptors [122]. *Bbs9* morphants display severe defects and lack outer segments [123].

Kinesins mediate the anterograde transport of IFT. In zebrafish, rod photoreceptors in *kif3a* and *kif3b* mutant fish degenerate rapidly, while cones undergo a slower degenerative process. The defects in photoreceptors are more severe in *kif3a* mutants compared to *kif3b* mutants [124,125]. Overexpression of *kif3c* in *kif3b* mutants can rescue photoreceptor defects, indicating functional redundancy between *kif3b* and *kif3c* [124]. The homodimeric kinesin Kif17 is essential for photoreceptor development. Knockdown of *kif17* disrupts outer segment formation and leads to mislocalization of opsin [126]. Retrograde IFT, which is essential for outer segment extension, is mediated by dynein. Knockdown of cytoplasmic dynein-2 in zebrafish results in smaller eyes and shorter photoreceptor outer segments [127].

Mutations in ciliary genes *CC2D2A*, *AHI1*, *ARL13B* and *CEP290* are the main causes of Joubert syndrome, characterized by abnormal brain development and retinitis pigmentosa [128–132]. Mutations in *cc2d2a* lead to unorganized outer segments and visual defects in zebrafish [133]. *Ahi1* mutants show shorter outer segments in cones at 5dpf in zebrafish, while the morphology and localization of rhodopsin in rods are not affected [134]. Zebrafish *arl13b* mutants exhibit shorter outer segments, and *arl13b* mutants display robust photoreceptor degeneration at 30dpf [135]. Loss of Cep290 results in severely disorganized outer segments in cones and photoreceptor degeneration at 1 year post-fertilization [136].

Defects in IFT, BBS proteins, kinesin and dynein have been implicated in photoreceptor degeneration, although the specific molecular mechanisms remain unclear. Several studies, including the work of Feng *et al.* [125], have indicated that mislocalization of opsin is a major contributor to photoreceptor degeneration. The accumulation of rhodopsin in the rod cell body membrane leads to rapid rod degeneration through downstream calcium signalling pathways in both *kif3a* and *kif3b* mutants. Further investigations revealed that the 44 C-terminal amino acids of rhodopsin are essential for its aggregation in the cell membrane. In *kif3a/rho* double mutants, the lack of the cytoplasmic tail of rhodopsin prevented rod degeneration [125]. Using proteomics and lipidomics approaches in zebrafish *bbs1* mutants, Masek *et al*. found that membrane-associated proteins, particularly those involved in lipid homeostasis, were enriched in the outer segment. This suggests an underlying pathomechanism of retinal degeneration in BBSome [119].

### 5.4. Cilia and spine

The spine plays a crucial role in vertebrates, providing structural support for the human body and protecting the nervous system. Among spinal disorders, scoliosis is the most common [137]. Scoliosis can be categorized into congenital scoliosis and idiopathic scoliosis, with the latter affecting approximately 2–3% of children and adolescents [138,139]. Idiopathic scoliosis, which accounts for about 90% of scoliosis cases, is characterized by three-dimensional curvature of the spine without obvious vertebral deformities. Its aetiology remains unknown, and it typically develops during adolescence, hence the term ‘adolescent idiopathic scoliosis' [140]. Scoliosis has become a major public health concern [140,141]. Understanding the aetiology of idiopathic scoliosis has been challenging due to the lack of suitable animal models [142,143]. However, in recent years, with advancements in gene editing technology and genomics, the mechanisms underlying scoliosis have started to be unraveled in zebrafish. Of particular interest is the association between abnormal cilia function and scoliosis [42,138,144–146].

The study of zebrafish *ptk7* mutants was the first to reveal the connection between abnormal cilia function and scoliosis [147]. This study demonstrated that a significant reduction in motile cilia on ependymal cells is the direct cause of scoliosis in zebrafish *ptk7* mutants. This reduction leads to the impaired directional flow of cerebrospinal fluid, which is normally driven by motile cilia [147]. The study further revealed that mutations in ciliary genes associated with PCD, such as *c21orf59*, *dyx1c1*, *ccdc151* and *ccdc40*, can cause scoliosis in zebrafish [147]. These findings confirm the importance of motile cilia in the ventricles for the normal development of the spine. It is suggested that motile cilia in the ventricles aid in the extension and diffusion of signalling molecules in the cerebrospinal fluid, which are essential for spinal development. Notably, Claire Wyart's group revealed that a fibre known as Reissner fibre is crucial for body axis development in zebrafish [148]. In zebrafish, Reissner fibre is a flexible fibre suspended in the cerebrospinal fluid, extending from the brain through the central canal to the tail. Defects in Sco-spondin, the main component of Reissner fibre, can lead to body axis bending and scoliosis in zebrafish [148,149]. Further research demonstrated that coordinated beating of motile cilia on the floor plate is necessary for the assembly of Reissner fibre [148]. Subsequent studies showed that cerebrospinal fluid-contacting neurons (CSF-cNs) on the floor plate are sensitive to the mechanical pressure of the cerebrospinal fluid (CSF). Loss of function in the ion channel protein Pkd2l1, which impairs the mechanical sensing ability of CSF-cNs, leads to mild scoliosis in zebrafish [150,151]. Exome sequencing analysis of families with idiopathic scoliosis revealed that genetic factors associated with cilia, microtubule skeleton, extracellular matrix, nerves and muscles may contribute to the development of scoliosis [152]. These studies collectively demonstrate the close relationship between cilia, cerebrospinal fluid, CSF-cNs and spinal development. However, the specific signalling molecules in the CSF that influence body axis development remain unclear.

In recent years, our group has also focused on understanding the mechanisms of body curvature caused by ciliary dysfunction in zebrafish [146]. By analysing zebrafish *zmynd10* mutants, which exhibit severe body curvature, we discovered that the flow of cerebrospinal fluid driven by ciliary movement is critical for maintaining body axis morphology [146]. Transcriptome analysis and drug screening revealed that dopamine signalling in the cerebrospinal fluid and Urotensin neuropeptides secreted by CSF-cNs have a significant impact on the normal development of the body axis in zebrafish. Futher findings suggest that motile cilia in the ventricles drive cerebrospinal fluid flow, and the dopamine signal in the cerebrospinal fluid promotes the secretion of Urotensin neuropeptides (Urp1 and Urp2) by CSF-cNs in the floor plate. Urotensin neuropeptides then bind to their receptor (Uts2r3) on dorsal muscle fibres to maintain the normal development of the body axis during early embryonic development. Intriguingly, mutations in Uts2r3 lead to severe scoliosis in adolescent zebrafish [146]. Furthermore, the spines of adult *urp1* mutants develop normally, while those of adult *urp2* mutants show slightly deformed curves [153]. However, *urp1/urp2* double mutants exhibit scoliosis similar to *uts2r3* mutants, suggesting functional redundancy between *urp1* and *urp2* [153]. These results further confirm the important functions of *urp1*, *urp2* and *uts2r3* in the development of the body axis in zebrafish [146,153]. Additionally, disruption of PCP in ependymal cells can also cause spinal curvature in zebrafish [42]. The urotensin signalling represents a key factor in maintaining spinal homeostasis in adult fish [42]. Strikingly, abnormal urotensin signalling also occurs in human idiopathic scoliosis patients, confirming the conservation of urotensin signalling in vertebrates [42].

Finally, recent studies have revealed that mutations in genes not directly involved in ciliary beating, such as *cep290*, *cc2d2a*, *bbs1*, *bbs5*, *kif6* and *kif7*, can also result in scoliosis in zebrafish [119,136,154–157]. These findings highlight the significance of cilia in spinal development. However, the molecular mechanisms underlying scoliosis caused by mutations in genes related to immotile cilia remain unclear. Further research is needed to elucidate the specific pathways and processes through which these genes contribute to spinal development and the development of scoliosis.

### 5.5. Cilia and kidney

Kidney plays a vital role in waste removal and maintaining homeostasis in animals. Disruption of kidney function due to environmental or genetic factors can lead to various kidney diseases, including acute kidney injury, kidney dysplasia, congenital abnormalities of the kidney and polycystic kidney disease (PKD). PKD, which includes autosomal dominant polycystic kidney disease (ADPKD) and autosomal recessive polycystic kidney disease (ARPKD), has been extensively studied. It is characterized by excessive proliferation of renal epithelial cells and the formation of fluid-filled cysts. ADPKD is the most common form of kidney disease, primarily caused by mutations in PKD1, which encodes Polycystin1 (PC1), and PKD2, which encodes Polycystin2 (PC2). The incidence rate of PKD in live births is estimated to be 1 in 400 to 1 in 1000 based on clinical surveys conducted in Denmark and the United States [158,159]. PC1 and PC2 are membrane proteins with 11 and 6 transmembrane domains, respectively [160,161]. The interaction between PC1 and PC2 forms a receptor-ion channel complex that regulates calcium ion levels in primary cilia [162]. Studies have shown that the polycystin 1/2 complex is in the ciliary membranes and responds to fluid flow by mechanically sensing calcium signals. Abnormalities in the ciliary structure or dysfunction of polycystins can disrupt the response to fluid flow in the kidneys, leading to cyst formation [163]. Although primary cilia are thought to respond to mechanical pressure in the kidney, the specific mechanisms by which primary cilia and polycystin complexes induce calcium ion signalling remain largely unknown [164,165].

Zebrafish kidneys are similar to those of mammals in terms of cell types and function. The transparency of embryos and growth *in vitro* make it convenient to observe the formation of polycystic kidneys under the microscope. Zebrafish have two *pkd1* genes: *pkd1a* and *pkd1b* [166,167]. *Pkd1a* mutants develop pronephric cysts starting at 2dpf, and by 3dpf, approximately 93% of the mutant embryos exhibit pronephric cysts. *Pkd1b* mutants, on the other hand, do not show pronephric cysts, but the expression level of *pkd1a* mRNA is significantly increased in the *pkd1b* mutants, indicating functional redundancy between *pkd1a* and *pkd1b* [168]. There is only one *pkd2* gene in zebrafish, and *pkd2* mutants do not exhibit pronephric cysts [169]. However, pronephric cysts can be induced by blocking maternal or zygotic *pkd2* mRNA using morpholinos [170]. In previously review, many researchers suggest that the absence of pronephric cysts in *pkd2* mutants may be due to the presence of *pkd2* maternal mRNA Previously [9]. Recently, we generated maternal *pkd2* mutants with PGC specifically conditional knockout (unpublished data). We do not observe pronephric cysts in these maternal *pkd2* mutant larvae. These results suggest that there may be other factors contributing to the absence of the pronephric cyst phenotype in *pkd2* mutants, and further investigation is needed.

Ciliary mutants in zebrafish often exhibit pronephric cysts. The motile cilia in the pronephric duct of zebrafish are crucial for proper kidney development, and defects in ciliogenesis, ciliary beating and cilia-mediated signalling can all lead to pronephric cysts [98,109,169,171,172]. Mutations in genes such as *ift57*, *ift81*, *ift88* and *ift172* have been shown to result in pronephric cysts [109,169,173]. Genes related to motile cilia, including *ccdc40* and *c21orf59*, can also contribute to the formation of pronephric cysts [98,174] . The PCP signalling pathway regulates the direction of cell division and the convergent extension process, which are crucial for the morphological development of the kidney duct. Abnormalities in PCP signalling, cell-oriented cell division (OCD) and convergent extension (CE) can lead to polycystic kidney [175,176]. Knockdown of *prickle1*, a gene involved in PCP signalling, in zebrafish has been shown to result in pronephric cysts [173,177]. Additionally, Cao *et al*. discovered that *inpp5e* plays a vital role in regulating the polarity of renal epithelial cells. *INPP5E* is a pathogenic gene associated with Joubert syndrome, a disorder that affects multiple organs and leads to cystic kidneys. However, the molecular mechanism underlying INPP5E dysfunction and its contribution to abnormal ciliogenesis and cystic kidneys remain unknown. Using zebrafish as a model, Cao *et al*. found that *inpp5e* mutations lead to pronephric cysts and defects in ciliogenesis. Further research revealed that *inpp5e* promotes ciliogenesis by regulating the polar distribution of PtdInd (4,5) P2 and PtdIns (3,4,5) P3 on the cell membrane [178,179].

Zebrafish offers significant advantages for drug screening in the context of polycystic kidney disease. Based on the dorsal curly phenotype observed in *pkd2* mutants or morphants, Cao *et al*. discovered that trichostatin A (TSA), a pan-HDAC (histone deacetylase) inhibitor, significantly reduced dorsal curling and the formation of pronephric cysts in *pkd2* morphants [180]. Metzner *et al*. found that inhibiting the activity of the *alk5* kinase with small molecule drugs effectively suppressed the dorsal curly phenotype in *pkd2* mutants, providing a foundation for further drug research [181]. Zebrafish is expected to play a crucial role in the screening and development of drugs for alleviating polycystic kidney disease.

### 5.6. Cilia and gematogenesis and fertilization

Motile cilia line the fallopian tubes and efferent ducts of the mammalian reproductive tracts, playing a pivotal role in the process of reproduction. Their primary functions include facilitating the transport of oocytes towards the uterus and moving sperm from testis into the epididymis [182,183]. Numerous studies have showed that genes mutation associated with ciliary motility or multiciliogenesis often resulting in male or female infertility. Notable examples of these genes encompass Gemc1, Mcidas, Ccno, miR-34b/c, miR-449, E2f4 and E2f5 [184–186]. The roles of cilia in fertilization have been extensively explored and discussed in several reviews. Furthermore, it is worth noting that sperm flagella share a conserved ultrastructural ‘9 + 2’ microtubular arrangement with motile cilia. Dysfunction in proteins associated with motile cilia often affects the formation or motility of sperm. Defects in sperm flagellar motility are typically linked to male infertility, a phenomenon observed in many patients with PCD [183,187].

Our current understanding of the physiological role of cilia within the reproductive system primarily comes from the study of motile cilia involvement in regulating mature gametes and fertilized. Nevertheless, it remains unclear whether cilia exist in germ cells and whether they contribute to gametogenesis. Gametogenesis encompasses the intricate process of producing sperm (spermatogenesis) and eggs (oogenesis) through meiotic division. It is widely acknowledged that primary cilia are transient structures that are generally incompatible with the cell division [188]. Maria *et al*. found that primary cilia persist stably through meiotic divisions in *Drosophila* spermatocytes, although their precise functions in this context remain unknown [189].

Recent research has shed light on the presence of cilia in zebrafish primary oocytes and primary spermatocytes during the first meiotic prophase [82,83]. Through immunofluorescence and three-dimensional imaging techniques, Mytlis *et al*. [83] reported the existence of zygotene cilium in zebrafish primary oocytes during the leptotene-zygotene stages of the first meiotic division. By analysing the *cep290*, *kif7* and *cc2d2a* zebrafish ciliary mutants, laser-induced excision of zygotene cilia, and live time-lapse imaging, their research revealed that absence of zygotene cilia in ciliary mutants disrupted the formation of chromosomal bouquet, the synaptonemal complex, germline cysts, ovarian development, as well as fertility. Importantly, they noted that the presence of zygotene cilia during meiosis is conserved in mammals [83]. Recently, López-Jiménez *et al*. performed a detailed structured and function analysis of cilia in mouse meiotic cells [190]. Additionally, Xie *et al*. detected the presence of cilia in both zebrafish primary spermatocytes and primary oocytes [82]. Their observations revealed that cilia emerged during the leptotene stage in primary spermatocytes and disassembled by the diakinesis stage. To decipher the function of cilia in spermatocytes, Xie *et al*. generated zebrafish mutants with PGC-specific conditional knockout of *kif3a*, a motor protein involved in anterograde transport. Their research revealed that the absence of *kif3a* led to impairments in cilia formation in both primary spermatocytes and primary oocytes. Consequently, this disruption triggered abnormalities in homologous recombination repair during the zygotene stage, ultimately resulting in diminished crossover formation and the apoptosis of spermatocytes. To further confirm the role of cilia in meiosis and eliminate any potential influence stemming from non-ciliary functions of Kif3a. Xie *et al*. constructed transgenic fish with heat shock-inducible *ift88* expression. Through a continuous regimen of heat shocking the *ift88* mutants and successfully rescuing them to adulthood, they provided additional compelling evidence that the loss of *ift88* function also led to spermatocyte apoptosis, thereby reinforcing the case for the involvement of cilia in meiotic process.

It is worth noting that all mutants in Mytlis's study [83] are global knockout, whereas in Xie's investigation, they generated germ-cell-specific conditional knockouts, effectively eliminating the possibility of cilia defects in other tissues. Xie *et al*. concluded that conditional knockout of cilia in germ cells does not significantly impede oogenesis and fertilization, a conclusion that finds reinforcement in earlier research by Borovina *et al*. [191]. Nevertheless, further investigations are required to dissect the precise molecular mechanisms underpinning the functions of cilia in gametogenesis.

## 6. Perspectives

Cilia are widely distributed in most human cells and tissues, and play crucial roles in organ development and haemostasis. Currently, it is estimated that there are more than 600 proteins associated with cilia, and mutations in ciliary proteins have been implicated in 35 known ciliopathies. A total of 187 genes have been identified as being associated with these ciliopathies, while an additional 241 candidate genes are yet to be fully explored [3,192]. However, the tissue specificity of cilia's physiological functions and the specific molecular mechanisms underlying ciliopathies remain largely unknown.

Zebrafish serves as a representative vertebrate organism for investigation of ciliary physiological function. In this paper, we provide an overview of latest research progress concerning the physiological function of cilia in zebrafish. Our review not only summarize some common and well-studied ciliary functions, such as their role in the establishment of left–right asymmetry, the maintenance of photoreceptor function, and kidney homeostasis but also sheds light on novel functions of cilia in vertebrate development, including their involvement in body axis and spine development, as well as their regulation of meiotic processes. Nonetheless, numerous vital scientific questions still require attention in these areas.

During the establishment of left–right asymmetry, cilia play a predominant role in detecting mechanical forces within the LRO. Nevertheless, recent data suggest that merely 20% to 30% of cilia in the LRO respond to oscillatory optical bending. This implies that the cilia in the LRO may exhibit heterogeneity, suggesting the existence of a distinct subpopulation of cilia with mechanical transduction capabilities [58]. Recent research in mice has shown that *pkd1l1* in the LRO is distributed asymmetrically through exosomes, affecting left–right asymmetry [193]. All these findings collectively suggest that the left–right asymmetry formation may be jointly regulated by mechanical and chemical signals in the LRO. These insights from recent studies highlight that exploring how cilia detect the external mechanical forces and influence the embryonic development in zebrafish is currently at the forefront of research.

In the development of photoreceptor cells, there are primarily two main issues to address. First, in ciliary mutants, the mechanism of photoreceptor degeneration caused by accumulation of opsin in the photoreceptor cell bodies remains unclear. Second, the mechanism governing the renewal of cone is currently not well understood. The development of cone cells in zebrafish is much closer to that in humans. Leveraging the benefits of transgenic technology, mechanisms such as the renewal of cone cells and the degeneration of photoreceptor cells will soon be elucidated.

Polycystic kidney disease in zebrafish embryos are mainly caused by motile cilia defects, which is different from that in humans. Furthermore, zebrafish *pkd1b* or *pkd2* mutants did not display polycystic kidney during the larval stage [168,169]. These results have significantly impacted the study of pathogenic mechanisms of polycystic kidney disease and, in particular, have hindered the possibility of conducting drug screening in zebrafish. Therefore, in the future, it is necessary to construct the conditional knockout mutants of *pkd1* or *pkd2* in adult zebrafish to conduct mechanistic studies.

The research of cilia regulate body axis and spine is currently a trending research topic. Although significant breakthroughs have been made in understanding the mechanism of cilia regulating the body axis and spine. There are still some unresolved issue that need clarification. For instance, the mechanism of spinal curvature caused by mutations in non-motile cilia genes requires further exploration. The coordinated movement of motile cilia and how they mediate the assembly of the Reissner fibre remain unclear. Additionally, the mechanism by which CSF-cNs in adult zebrafish transmit Urotensin neuropeptide through dense tissue to muscle fibres remains unexplained. Future technological innovations will help address these complex questions.

Ciliary regulation of meiosis is a new field. There is controversy regarding whether the primary cilia on primary oocytes or primary spermatocytes regulate meiosis by influencing the spatial structure of chromosomes during the synaptonemal complex stage or through signal transduction. Additionally, the specific impact of the cilia on primary spermatocytes or primary oocytes on meiosis and gametogenesis remains unclear. Conditional disruption of ciliary growth in the zebrafish germline, in combination with the latest techniques, such as single-cell sequencing, ATAC sequencing and CUT&Ttag sequencing, will contribute to addressing these scientific questions.

From the perspective of cilia research in vertebrates, future trends will focus on the following aspects: (1) discovering new types of cilia; (2) revealing new functions of cilia; (3) further elucidating the ultrastructure of cilia; (4) establishing cilia-related animal disease model; and (5) conducting drug screenings for ciliary disease. Currently, using zebrafish as a model organism has advantages in all the aspects mentioned above, except for the third. First, further observation is required to determine whether there are yet undiscovered cilia in the complex tissues and organs of zebrafish. Second, the cilia already discovered in zebrafish provide opportunities to uncover the physiological functions of cilia in other tissues, such as the skin, muscle fibres, the notochord and hypochord. It requires further exploration to understand the functions of cilia in these tissues. Moreover, the trend of combining clinical data analysis with disease model construction in ciliopathy research is becoming more and more popular. Constructing single-base mutations found in certain diseases in zebrafish and tissue-specific knockouts will become the preferred approach for building disease models. Lastly, given the genetic pleiotropy and heterogeneity of ciliopathies, future research should centre on employing structural biology and artificial intelligence to investigate mechanisms not only at the genetic level but also down to the granularity of individual amino acid residues. The combined use of these methods will provide a broader outlook for drug screening using zebrafish as a model.

## Data Availability

All data generated or analysed are included in this article.
